# Methylene blue-loaded niosome: preparation, physicochemical characterization, and in vivo wound healing assessment

**DOI:** 10.1007/s13346-020-00715-6

**Published:** 2020-02-25

**Authors:** Ali Farmoudeh, Jafar Akbari, Majid Saeedi, Maryam Ghasemi, Neda Asemi, Ali Nokhodchi

**Affiliations:** 1 Department of Pharmaceutics, Faculty of Pharmacy, Mazandaran University of Medical Sciences, Sari, Iran; 2 Department of Pathology, Faculty of Medicine, Mazandaran University of Medical Sciences, Sari, Iran; 3 Analytical division, Faculty of Chemistry, University of Mazandaran, Babolsar, Iran; 4 Pharmaceutics Research Laboratory, School of Life Sciences, University of Sussex, Brighton, BN1 9QJ UK; 5 Drug Applied Research Center and Faculty of Pharmacy, Tabriz University of Medical Sciences, Tabriz, Iran

**Keywords:** Niosomal gel, Methylene blue, Box–Behnken design, Full-thickness wound model, Wound healing, Malondialdehyde, Superoxide dismutase

## Abstract

Following skin injury, the overproduction of reactive oxygen species (ROS) during the inflammatory phase can cause tissue damage and delay in wound healing. Methylene blue (MB) decreases mitochondrial ROS production and has antioxidant effects. The authors aimed to prepare MB-loaded niosomes using the ultra-sonication technique as a green formulation method. A Box–Behnken design was selected to optimize formulation variables. The emulsifier to cholesterol ratio, HLB of mixed surfactants (Span 60 and Tween 60), and sonication time were selected as independent variables. Vesicle size, zeta potential (ZP), and drug entrapment capacity percentage were studied as dependent variables. The optimized formulation of niosomes showed spherical shape with optimum vesicle size of 147.8 nm, ZP of − 18.0 and entrapment efficiency of 63.27%. FTIR study showed no observable interaction between MB and other ingredients. In vivo efficacy of optimized formulation was evaluated using an excision wound model in male Wistar rat. Superoxide dismutase (SOD, an endogenous antioxidant) and malondialdehyde (MDA, an end product of lipid peroxidation) levels in skin tissue samples were evaluated. After 3 days, MDA was significantly decreased in niosomal gel-treated group, whereas SOD level was increased. Histological results indicate rats that received niosomal MB were treated effectively faster than other ones.

**Electronic supplementary material:**

The online version of this article (10.1007/s13346-020-00715-6) contains supplementary material, which is available to authorized users.

**Figure:**
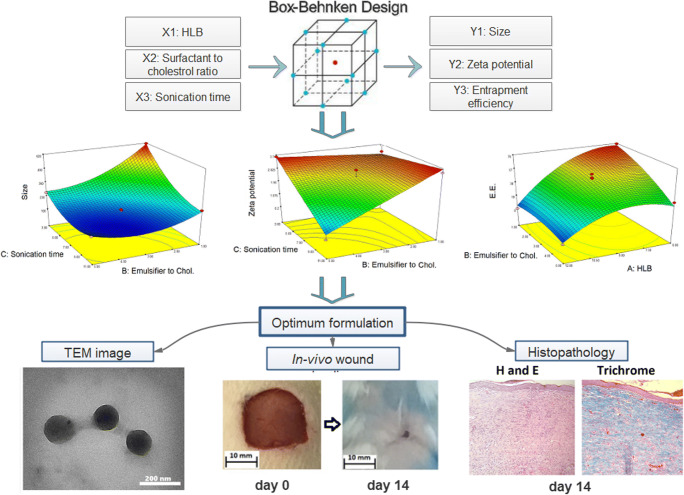
Graphical abstract

## Introduction

The cutaneous wound healing process is divided into four predictable phases: hemostasis, inflammation, proliferation (growth of new tissue), and maturation (tissue remodeling) [1]. The hemostatic phases involve platelet aggregation and fibrin clot formation. After hemostasis, inflammatory cells (neutrophils and monocytes) migrate into the injured tissue. These cells play an important role in defense against invasive microorganisms and secrete growth factors that are required for the proliferation phase [1, 2]. Inflammatory cells are important sources of reactive oxygen species (ROS) that can induce damage to the surrounding tissues by cross-linking of proteins and peroxidation of lipids [3]. Recent studies have highlighted that low levels of ROS are required for cell signaling and angiogenesis [4]. Thus, ROS are important in the wound healing process, but the overproduction of these molecules causes oxidative stress, resulting in prolonged and impaired wound healing. Therefore, the balance between production and elimination of ROS is necessary for the normal healing process. The defense system against overexpression of ROS is achieved by a variety of endogenous antioxidant molecules Including superoxide dismutase glutathione peroxidase, and catalase [5]. There are also exogenous antioxidant compounds that accelerate wound healing processes like vitamin E, ascorbic acid, and phenolic compounds [4].

Methylene blue (MB) is a phenothiazine dye with a redox potential of 11 mV. Because of its low redox potential, MB molecule can switch between its reduced and oxidized forms (MB and MBH_2_ respectively), which facilitates mitochondrial electron transport and results in reduced ROS production [6]. Recent studies have shown that MB increases the longevity of skin fibroblasts and improves cell proliferation; thus, it could promote wound healing procedure [7]. Rosique et al. studied burn wound healing in an animal model and showed that treatment with MB reduces the progression of burn and accelerates skin recovery [8] but its efficacy in the surgical wound healing process has not yet been studied. MB is an oxygen-sensitive compound [9] and must be protected against O_2_ when it is used in a transdermal formulation. In recent decades, the potential of niosomes as drug delivery systems has been extensively studied [10–12]. Niosomes are colloidal lamellar vesicles formed by mixing cholesterol (CHOL) and nonionic surfactants [13]. The unique structures of these vesicles make them able to encapsulate both hydrophobic and hydrophilic drugs [14]. Niosomes can protect the encapsulated drug from environmental degradation and prolong its half-life during blood circulation [15]. The flexibility of these vesicles allows them to penetrate into the deep layers of the skin [16, 17].

The purpose of the current study was to design and develop an effective MB niosome formulation using a Box–Behnken statistical design and evaluate the vesicle size, zeta potential (ZP), entrapment efficiency (EE), and in vitro drug release kinetics. Also, the healing effect of MB niosome was evaluated using a full-thickness wound model.

## Materials and methods

### Materials

MB was purchased from Sigma–Aldrich (USA). Sorbitan monostearate (Span 60), Polysorbate 60 (Tween 60), sodium hydroxide (NaOH), potassium dihydrogen phosphate (KH_2_PO_4_), and Sodium carboxymethyl cellulose were purchased from Merck KGaA (Germany). Cholesterol was purchased from Solar Bio Chemicals (China). Lipid peroxidation and superoxide dismutase assay kits from Navand Salamat Company (Urmia, Iran). All chemicals, used in the study were of analytical grades. Fresh deionized water was obtained from the Human Ultra-Pure System (Human Corp, Korea).

### Niosome preparation by ultra-sonication technique

Niosomes were prepared by direct ultra-sonication of samples. Briefly, 5 mg of MB dissolved in 20 ml of hot deionized water (80 °C) and the solution was added to a pre-heated mixture of surfactants and cholesterol. After hydrating the mixture for 60 min, the hot distilled water was gradually added to the system and the final volume reached 100 ml. After 20 min stirring at 500 rpm, the system was sonicated using a probe sonicator (SONOPLUS mini20, Bandelin electronic, Berlin, Germany) in pulsed mode (45 s pulse on and 15 s off, amplitude 50%). Then, the prepared niosomes were kept in the fridge (4–8 °C) for further characterization.

### Experimental design

In this study, a 3-factor, 3-level Box–Behnken statistical design with 17 experiments was employed to optimize the formulation variables (Table 1). The independent variables were emulsifier to CHOL ratio (X1), HLB of mixed surfactants (Span 60 and tween 60) (X2) and sonication time (X3). The EE percent (Y1), drug loading percent (DL%) (Y2), vesicle size (Y3), and ZP (Y4) were selected as dependent variables. The nonlinear quadratic model generated by the design is shown below:

$$ Y={\mathrm{b}}_0+{\mathrm{b}}_1{\mathrm{X}}_1+{\mathrm{b}}_2{\mathrm{X}}_2+{\mathrm{b}}_3{\mathrm{X}}_3+{\mathrm{b}}_{12}{\mathrm{X}}_1{\mathrm{X}}_2+{\mathrm{b}}_{13}{\mathrm{X}}_1{\mathrm{X}}_3+{\mathrm{b}}_{23}{\mathrm{X}}_2{\mathrm{X}}_3+{\mathrm{b}}_{11}{{\mathrm{X}}_1}^2+{\mathrm{b}}_{22}{{\mathrm{X}}_2}^2+{\mathrm{b}}_{33}{{\mathrm{X}}_3}^2 $$

where *Y* is a response (dependent variable), X1, X2, and X3 are the independent variables and b_0_ is the measured intercept; *b*_*i*_, *b*_*ij*_, and *b*_*i*_^*2*^ are regression coefficients for the main effects (linear effect), two-way interactions, and quadratic effect, respectively. The adequacy of mathematical models was evaluated using the lack-of-fit test. The experimental data were modeled using the Design–Expert software version 7.0.0 (Stat-Ease Inc.; Minneapolis, MN, USA).

**Table 1:** Experimental runs and averaged observed responses for BBD (data shown as mean ± standard deviation, *n* = 3)

Run	Independent variables			Responses			
	X1	X2	X3	Y1	Y2	Y3	Y3
				Mean ± SD	Mean ± SD	Mean ± SD	Mean ± SD
1	9	3	7	10.38 ± 2.33	151.46 ± 4.05	46.08 ± 2.91	1.12 ± 0.07
2	9	5	11	1.32 ± 0.68	182.56 ± 4.77	24.28 ± 1.36	0.40 ± 0.02
3	12	1	7	20.60 ± 3.84	270.80 ± 2.98	0.19 ± 0.33	0.01 ± 0.02
4	9	1	3	24.59 ± 4.26	613.76 ± 9.42	48.92 ± 3.24	2.41 ± 0.16
5	6	5	7	6.39 ± 2.51	156.16 ± 5.56	31.35 ± 4.16	0.52 ± 0.07
6	12	3	3	21.53 ± 3.39	417.16 ± 12.50	0.00 ± 0.00	0.00 ± 0.00
7	9	3	7	12.29 ± 2.89	110.83 ± 4.65	45.30 ± 3.06	1.12 ± 0.08
8	9	1	11	30.65 ± 2.78	248.60 ± 17.61	51.48 ± 2.72	2.54 ± 0.13
9	12	3	11	9.82 ± 5.27	147.16 ± 3.52	5.51 ± 0.99	0.13 ± 0.02
10	6	3	11	15.66 ± 2.63	117.83 ± 5.57	43.18 ± 5.09	1.07 ± 0.13
11	9	3	7	23.56 ± 5.59	176.23 ± 3.47	52.57 ± 2.60	1.30 ± 0.06
12	6	3	3	34.70 ± 6.70	317.73 ± 4.41	41.89 ± 3.93	1.04 ± 0.10
13	6	1	7	33.53 ± 3.89	417.03 ± 19.10	75.22 ± 2.33	3.71 ± 0.11
14	9	3	7	11.51 ± 3.72	173.13 ± 13.56	54.39 ± 4.18	1.35 ± 0.10
15	9	5	3	35.96 ± 5.77	264.26 ± 5.35	22.45 ± 3.86	0.37 ± 0.06
16	9	3	7	14.90 ± 2.01	156.20 ± 2.69	59.26 ± 3.63	1.47 ± 0.09
17	12	5	7	11.40 ± 3.56	177.90 ± 7.15	0.31 ± 0.54	0.01 ± 0.01

Emulsifier to CHOL ratio (*X1*), HLB of mixed surfactants (*X2*), sonication time (*X3*), encapsulation efficiency percent (*Y1*), vesicle size (*Y2*), zeta potential (*Y3*)

### Characterization of MB-loaded niosomes

The mean size, polydispersity index (PDI) and ZP of niosomes were analyzed by Dynamic light scattering (DLS) using the Zetasizer Nano-ZS device (Malvern Instruments Ltd., UK). The diffraction pattern (XRD) was obtained using an X-ray diffractometer (model D8-Advance, Bruker AXS, Germany) at 40 kV, 30 mA, the angle range of 4 to 45° at a scan speed of 1°/min. FTIR spectroscopy was performed to characterize the interaction between the components. Infrared spectra were recorded in the range of 4000–450 cm^−1^, and the resolution of 1 cm^−1^ using an FTIR-One spectrometer (PerkinElmer, USA). Differential scanning calorimetry (DSC) measurements were carried out using a differential scanning calorimeter model pyris6 (PerkinElmer, Norwalk, USA). Nano-vesicles morphological state was examined using an EM 208S transmission electron microscope (TEM, Philips, Netherlands).

### Encapsulation efficiency

The amount of encapsulated MB in the niosomal vesicles was evaluated using the centrifugation method. The niosomal dispersion was centrifuged at 27,000 rpm at 4 °C for 60 min (SIGMA 3-30KS refrigerated centrifuge, Germany). Then, the supernatant was collected and quantified by a UV-Vis Spectrophotometer (Jasco V-630, Japan) at 664 nm. The amount of entrapped drug (EE %) was calculated using the following equation:

$$ \mathrm{E}.\mathrm{E}.\%=\frac{\mathrm{Encapsulated}\ \mathrm{drug}\ \mathrm{weight}}{\mathrm{Total}\ \mathrm{drug}\ \mathrm{weight}}\times 100 $$

The drug loading percent (D.L.%) of the formulations was also calculated, showing the relationship between the weight of the entrapped drug and the total weight of the niosome components.

$$ \mathrm{D}.\mathrm{L}.\%=\frac{\mathrm{Encapsulated}\ \mathrm{drug}\ \mathrm{weight}}{\mathrm{Total}\ \mathrm{niosome}\ \mathrm{weight}}\times 100 $$

### Preparation of niosomal gel formulation

The optimized formulation was prepared and centrifuged (1 h, 27,000 rpm at 4 °C). Then the Sediment was redispersed in 1.5 ml deionized water and centrifuged for another 15 min to purify nano-vesicles. In the last step by lyophilizing the sediment, powdered MB niosomes were obtained. To prepare the niosomal gel, the freeze-dried powder of optimum formulation was used. The colloidal dispersion was frozen in liquid nitrogen for 10 min and dried using a freeze-drier apparatus (Christ Alpha 1–2 LD plus, Martin Christ, Germany). The amount of the dried powder equivalent to 5 mg of MB was dispersed in 100 ml of distilled water. Then, sodium carboxymethyl cellulose (2 g) was added as a gelling agent. The mixture was left overnight at 4 °C, then stirred to obtain a homogeneous gel (0.005% w/w).

### In vitro drug release study

The dialysis tube diffusion technique was used to determine the release profile of MB from niosomal dispersion, free drug solution, and Na-CMC gel containing each of them. After centrifugation (1 h, 27,000 rpm at 4 °C), the sediment was separated and redispersed in the same volume of deionized water. Then 5 ml of dispersion was placed in the dialysis tube, hermetically sealed and soaked in a tube containing 35 ml of phosphate buffer (pH 5.5). The entire system was kept at 32 °C ± 0.5 °C with continuous shaking using an incubator shaker (KS 4000 ic control, IKA, Germany). The same tests were performed for free drug solution and gel formulations. Aliquots of the sample were withdrawn from the receiver solution at predetermined time intervals followed by filtering and analyzing the drug release using a spectrophotometer at 664 nm. After each assay, the sample was returned to the receiver container. For all dissolution in vitro tests, the sink condition was maintained.

### Drug release kinetics

Drug release kinetics were evaluated using Kinet DS software version 3.0 (Jagiellonian University Medical College, Poland). In vitro drug release data were fitted to four mathematical models (zero order, first order, Weibull and Korsmeyer–Peppas). The determination coefficient (r^2^) for all models was calculated to find the best fitting model.

### Animal models

Male Wistar rats (weighing 200 to 250 g; laboratory animal center of Mazandaran University of medical science, MAZUMS, Mazandaran, Iran) were used in this study. The animals were housed individually at a constant temperature of 22 °C and 12 h light/dark cycle. They allowed free access to water and food during the period. All animal procedures based on the Guideline for the Care and Use of Laboratory Animals prepared by National Institutes of Health and were approved by the Animal Research Committee of MAZUMS (IR.MAZUMS.REC.1397.1373).

### Full-thickness wound model

To create short-term anesthesia, xylazine (20 mg/kg) and ketamine (100 mg/kg) were diluted in 100 μl of normal saline and injected intraperitoneally. The dorsal hairs were shaved and the skin of the area was sterilized with ethanol (70%). Full-thickness surgical excisions were performed on rat dorsal trunk skin by cutting a 2 × 2 cm area.

After surgery, rats were randomly divided into three groups of 10. Then the animals were randomly grouped as follows, group I-niosomal MB (0.005% w/w), group II- simple MB gel (0.005% w/w), and group III-controlled group that received gel without the active ingredient. Two milliliters of gel formulations were applied topically once a day for 3 weeks to each group.

### Rate of healing

The wound healing progression was assessed by digital photography. The healing rate was calculated by Gilman et al. [18, 19]. In this method, the wound area and the wound circumference are measured and the linear healing rate (D) is calculated according to the following formula:

$$ \mathrm{D}=\frac{\mathrm{Wound}\ \mathrm{size}\ \mathrm{at}\ \mathrm{t}\mathrm{ime}\ \mathrm{t}2-\mathrm{Wound}\ \mathrm{size}\ \mathrm{at}\ \mathrm{t}\mathrm{ime}\ \mathrm{t}1}{\mathrm{Pavg}\times \left(\mathrm{t}2-\mathrm{t}1\right)} $$

In this equation, P_avg_ is the mean of the wound circumference at times t1 and t2.

### Histology

Three rats in each group were sacrificed on postoperative days 3, 7, and 14 for histopathological and enzymatic examinations. Tissue samples from the wound area were removed by surgical excision and immediately fixed in a 10% formalin solution. Then these samples were embedded in paraffin blocks and 4–7 μm slices were prepared. All samples were sectioned and stained with hematoxylin and eosin (H&E) and Masson’s trichrome (MT). Stained sections were observed for the pathological changes using a light microscope. In this study, a scoring system was used as a semi-quantitative method to compare wound healing speed between groups. A number of 6 histological factors were evaluated including epidermal regeneration, amount of granulation tissue, inflammatory cells infiltration, Angiogenesis, Proliferation of fibroblast cells, and Collagen deposit [20]. Factors scored from 0 to 4 and the presence of normal structures received the highest scores.

### Lipid peroxidation and antioxidant activity in granulation tissue

As mentioned earlier, three animals of each group were sacrificed on days 3, 7, and 14. Then, four pieces of 5-mm skin punch biopsy samples were taken at a distance of 2–3 mm from the wound margin. After rinsing with cold physiological saline and potassium chloride (1.15% KCl), the tissue samples were homogenized in potassium phosphate buffer (KPB, 0.1 M) and centrifuged at 18000 rpm for 20 min to remove debris. The clear supernatant was stored in − 70 °C until antioxidant enzyme assay.

### Lipid peroxidation

Malondialdehyde (MDA) was assayed as an end product of lipid peroxidation in tissue homogenate using an MDA Colorimetric Assay Kit using the thiobarbituric acid method [21]. Briefly, 200 μL of tissue homogenate was mixed with 800 μL of 0.67% thiobarbituric acid (TBA) and heated on a water bath at 95 °C for 45 min, then cooled under the tap. The absorbance of the sample was then measured spectrophotometrically at 550 nm. Then UV absorbance data of samples were interpolated from the standard curve to calculate the concentrations of MDA in tissue. The amount of MDA was expressed as nanomoles of MDA formed per gram of skin tissue.

### Superoxide dismutase (SOD) activity

The total superoxide dismutase (SOD) activity of the supernatants was assayed based on the inhibition of pyrogallol autoxidation according to Marklund [22]. The SOD activity was measured by using an assay kit (Navand Salamat Company, Urmia, Iran). The reaction mixture and blank were prepared following the manufacturer’s instructions and the enzyme activity was carried out at 420 nm for 3 min against the blank and was calculated as follows:

$$ \mathrm{SOD}\ \mathrm{activity}\ \left(\frac{\mathrm{U}}{\mathrm{gram}}\right)=\frac{\Delta \mathrm{OD}\ \mathrm{Control}-\Delta \mathrm{OD}\ \mathrm{Test}}{\Delta \mathrm{OD}\ \mathrm{Control}}\times \frac{\mathrm{Vs}}{\mathrm{Vt}}\times \frac{\mathrm{D}}{\mathrm{m}} $$

To calculate ΔOD_control_ the difference in absorbance of the control sample was measured every 60 s for 3 min, then the average was calculated. The same calculations were performed for the test samples (ΔOD _test_). In the above equation, Vs is the total volume of the sample solution and Vt is the volume of sample used for testing. Other factors are m and D which are respectively attributed to the weight of the solid sample in gram and dilution factor.

## Results and discussion

### Effect of independent factors on EE and physical characteristics of MB Niosomes

#### Effect of independent variables on EE

EE percent showed a wide variation from a minimum of 0% to a maximum of around 75% (Table 1). Niosomes consist of an aqueous core surrounded by one or more bilayers composed of nonionic surfactants. The extent of drug entrapment in vesicles is influenced by the physicochemical properties of the drug, especially the partition coefficient (logP). MB with intermediate Log *P* values of 0.9 partitions between the bilayer and in the aqueous core [23]. The effect of nonionic surfactants on the drug entrapment efficiency depends on their concentration [14], as well as HLB when two or more surfactants are added to the formulation [24]. To derive a relation between independent factors and EE percent of niosomes, different mathematical models were examined using the Box–Behnken design. The quadratic model showed a good fit of the experimental data with a *P* value of 0.0001 and lack of fit P value was 0.4866.

$$ \mathrm{E}.\mathrm{E}.\%=51.52-23.20\times \mathrm{A}-12.18\times \mathrm{B}+1.40\times \mathrm{C}+11.00\times \mathrm{A}\times \mathrm{B}+1.06\times \mathrm{A}\times \mathrm{C}-0.18\times \mathrm{B}\times \mathrm{C}-19.45\times {\mathrm{A}}^2-5.31\times {\mathrm{B}}^2-9.43\times {\mathrm{C}}^2 $$

**Fig. 1:**
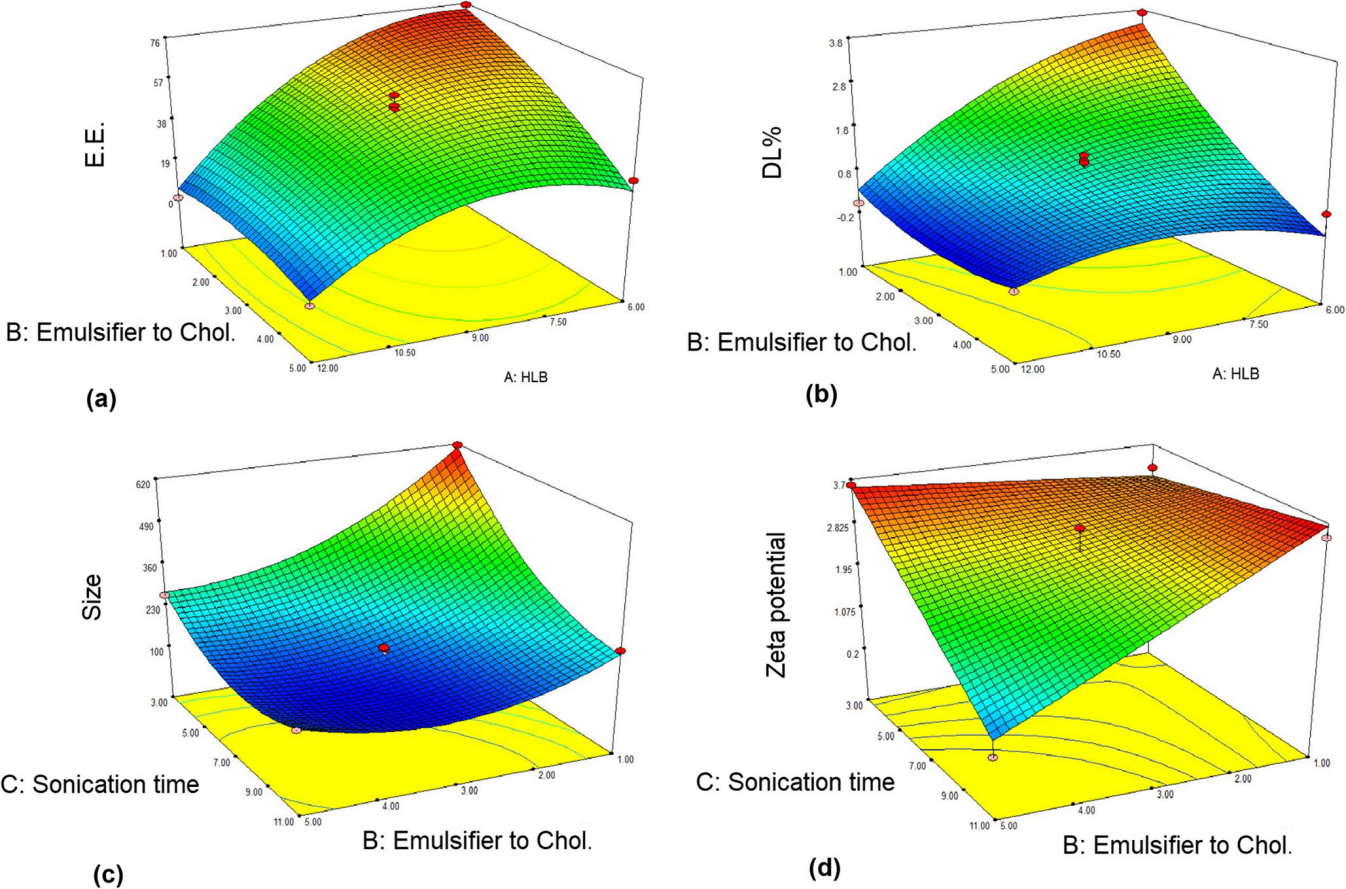
Response surface 3D plots for the effect of independent variables on responses (**a** encapsulation efficiency percent (E.E.%), **b** drug loading percent (DL%), **c** particle size, and **d** zeta potential (ZP))

The model showed a proportional pattern between the HLB value (A) and the EE (*P* < 0.05). As the HLB decreased, EE percent of the niosomes increased (Fig. 1a). This may be due to the chemical structure of surfactants. Span 60 with a smaller head group (sorbitan) as compared to Tween 60 (polyoxyethylene) decreased fluidity and permeability of bilayer; this in turn can improve the drug entrapment [25, 26]. Increasing the amount of cholesterol in the formulation improves membrane rigidity; thus, increasing the emulsifier to cholesterol ratio (B) has a negative effect on drug entrapment (*P* < 0.05) (Fig. 1a). Formation of hydrogen bonds between the 3 beta-hydroxy groups of the cholesterol and the oxygen at the sorbitan monostearate ester group resulting in highly ordered gel formation, leading to less leaky and higher EE [27]. FTIR results confirmed the possibility of hydrogen bonding between them which will be discussed later in the manuscript. According to the mathematical model, the interaction effect of A and B was found to give a positive impact on %EE (*P* < 0.05). Varying the sonication time and independent factor interaction affected the EE percent, but the changes were not statistically significant. DL% was also calculated for all formulations. This parameter shows the amount of drug loaded per unit weight of niosomes, and the results showed that DL was varied between 0 and 3.71 ± 0.11%. The relationship between the DL% and the independent variables is shown in the following mathematical model.

$$ \mathrm{DL}\%=+1.20-0.77\ \mathrm{A}-0.92\ \mathrm{B}+0.80\ \mathrm{A}\ \mathrm{B}-0.55\ {\mathrm{A}}^2+0.32\ {\mathrm{B}}^2 $$

The quadratic model showed a good fit to the DL% data but lack of fit test was significant (*P* > 0.05). To solve this problem, backward regression was used to remove the minimum number of non-significant model terms. In the modified model the *p* value was 0.0001 and lack of fit was 0.056. The correlation pattern between DL% and independent variables was similar to E.E%. This equation shows that as the variables A and B increased, the percentage of DL decreased (*P* < 0.05). Also, the interaction between these two variables had a significant and negative effect on drug loading percentage (P < 0.05) (Fig. 1b).

#### Effect of independent variables on vesicle size

Table 1 shows that the size of the prepared vesicles ranged from 110.88 ± 4.65 nm to 613.76 ± 9.41 nm. The relationship between independent variables and the size of vesicles is presented as follows:

$$ \mathrm{Size}=153.57+0.53\ \mathrm{A}-96.16\ \mathrm{B}-114.60\ \mathrm{C}+41.99\ \mathrm{A}\mathrm{B}-17.52\ \mathrm{A}\mathrm{C}+70.87\ \mathrm{B}\mathrm{C}+12.29\ {\mathrm{A}}^2+89.61\ {\mathrm{B}}^2+84.11\ {\mathrm{C}}^2 $$

The quadratic model showed a good fit of the experimental data with a *p* value of 0.0004 and the lack of fit *p* value was 0.1. The results indicated that vesicle size decreased with an increase in cholesterol to surfactant ratio (B) and sonication time (C) (*p* < 0.05) which is in agreement with previous findings [28–30]. Also, the interaction between A and B had a positive effect on the particle size (*p* < 0.05) (Fig. 1c).

#### Effect of independent variables on ZP

Table 1 shows that ZP varied from 1.32 ± 0.68 mV to 35.96 ± 5.77 mV and all the formulations exhibited negative charges. Emulsifier to Chol (B) ratio and sonication time (C) are the most significant factors affecting the ZP of the vesicles (*p* < 0.05). The equation to predict the ZP of niosomes can be expressed as:

$$ \mathrm{Ln}\left(\mathrm{ZP}\right)=+2.71-0.11\times \mathrm{A}-0.63\times \mathrm{B}-0.58\times \mathrm{C}+0.27\times \mathrm{A}\times \mathrm{B}+2.657\times \mathrm{A}\times \mathrm{C}-0.88\times \mathrm{B}\times \mathrm{C} $$

The 2-factor interaction (2FI) model showed a good fit of the experimental data with a *p* value of 0.0002 and the lack of fit *p* value was 0.495. The model showed that decreasing sonication time and increasing the cholesterol/ emulsifier ratio resulted in a significant increase in the electrical charge intensity (*p* < 0.05). Also, the interaction of B and C had a significant effect on the particle size reduction (*p* < 0.05) (Fig. 1d). Previous studies have shown that CHOL has a negative surface charge in the pH range 2.0–10.5 [31, 32]. On the other hand, nonionic surfactants and MB have no electrical charge; therefore, the electrical surface charge of the vesicle depends only on the amount of cholesterol.

### Optimization

The point prediction method was applied to the selected optimized MB-loaded niosomal formulation for attaining the minimum vesicle size and maximum value of EE. The optimal values of independent variables were HLB 7.36, emulsifier to cholesterol ratio of 2.62, and sonication time of 8.6 min. The predicted optimized formulation had the vesicle size of 148.99 ± 18.19 nm with ln(ZP) of 2.75 ± 0.12 (ZP average = 15.67) and EE of 60.51 ± 2.64% whereas the values of the observed response were found to be 147.8 nm, − 18.0 mV, and 63.27% respectively (Supplementary Fig. 1). There was a linear relationship between predicted and experimental values of all dependent variables that indicate the selected models fitted to the experimental data very well (Supplementary Fig. 2).

### Physical properties of optimized vesicles

**Fig. 2:**
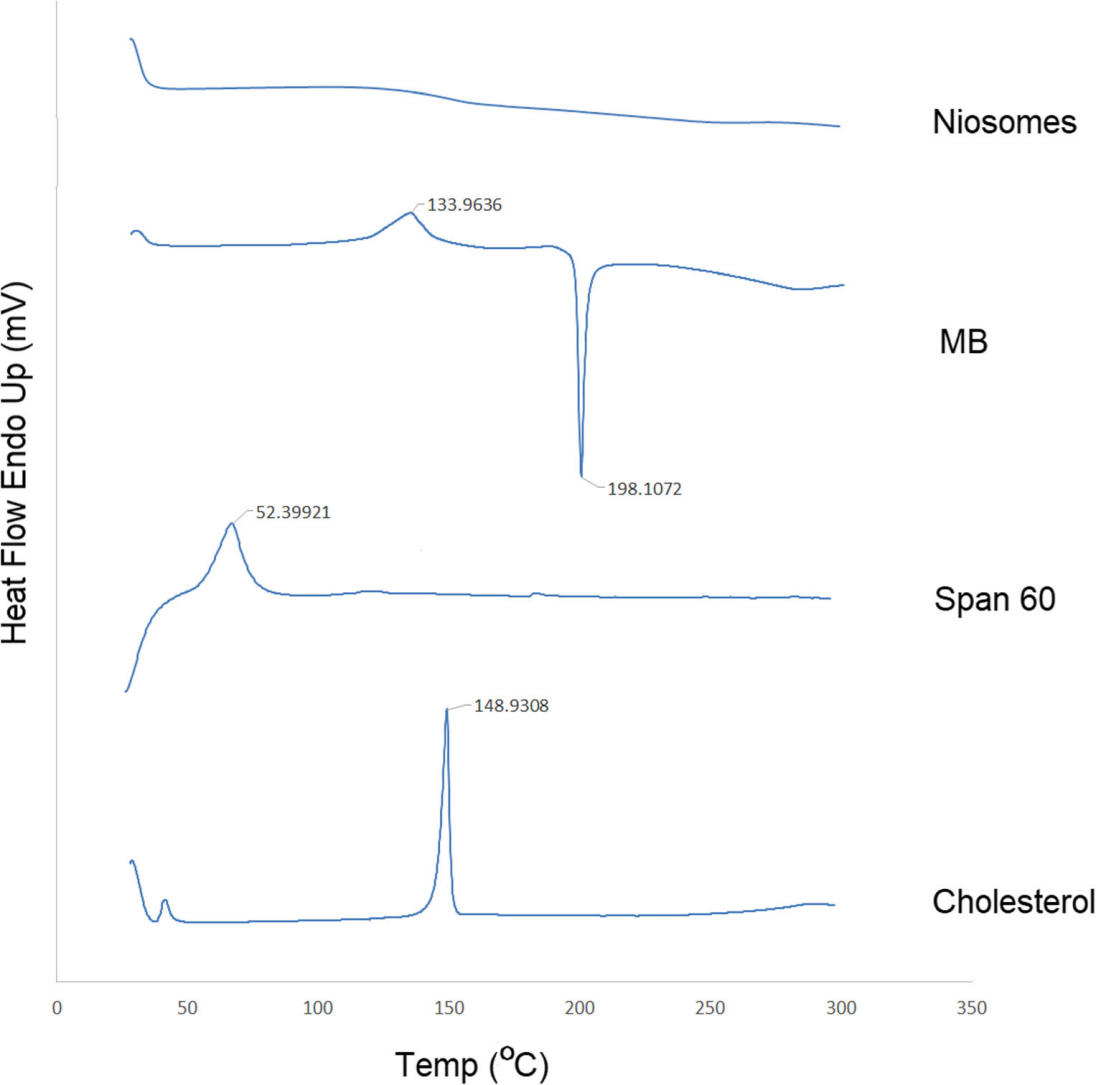
Differential scanning calorimetric thermograms of MB, Span 60, cholesterol, and freeze-dried niosome powder

The DSC and XRD tests were used to investigate the crystalline structure of MB and excipients in niosomal formulation. The thermographs for MB, Span 60, cholesterol and freeze-dried niosome powder are illustrated in Fig. 2. The DSC scan of Span 60 and cholesterol displayed a single endothermic melting peak at 52.399 and 148.930 °C, respectively. DSC curve of MB exhibited an endothermic pick at 133.963 and an exothermic pick at 198.107 °C. Previous studies on the crystalline state of MB demonstrated the presence of methylene blue in five different hydrate forms, so the endotherm could be attributed to solid state transformation of two hydrate forms and the presented exotherm shows MB decomposition [33].

**Fig. 3:**
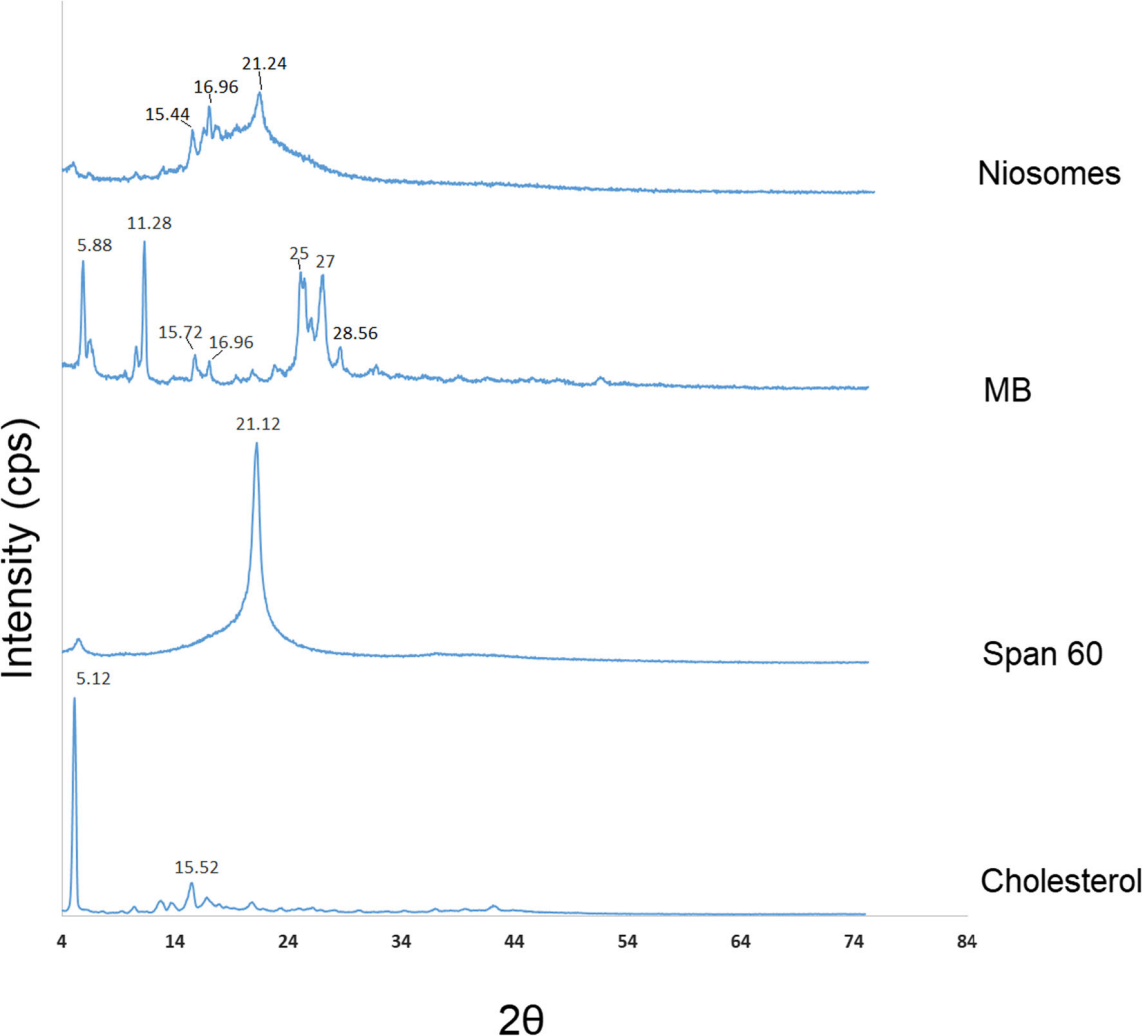
The Powder X-ray spectra of freeze-dried niosome powder, MB, Span 60, and cholesterol

Niosomal sample spectrum showed no observable peak suggesting MB and other crystalline ingredients were present in the amorphous state. The XRD spectra of freeze-dried niosome powder, MB, Span 60, and cholesterol are shown in Fig. 3. Pure MB powder exhibited numerous sharp peaks at 5.88°, 11.28°, 25°, and 27°. Span 60 showed a single peak at 21.12° and cholesterol exhibited intense peaks at 5.12° and 15.52°. XRD diffraction of free MB and other ingredients confirmed their crystalline nature and the diffraction patterns were in good agreement with the data reported in previous studies [34–36]. Niosome spectra presented fused peaks with few separated peaks (almost all characteristic peaks were disappeared), which indicates the amorphous structure of the drug and excipients. These results are in accordance with the DSC data.

**Fig. 4:**
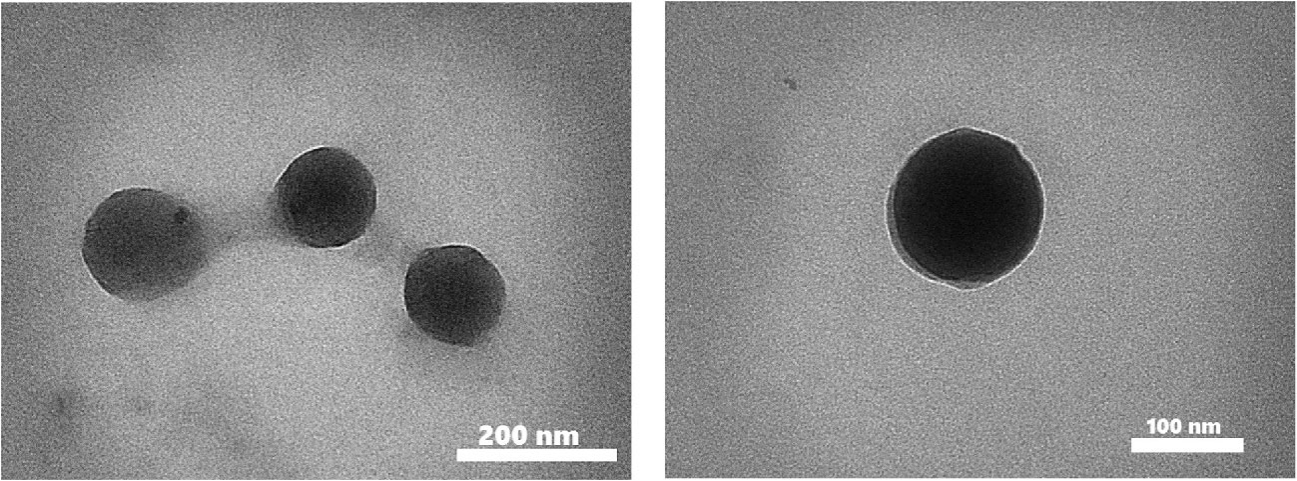
TEM images of MB niosomes

TEM analysis has demonstrated the presence of spherical vesicles in the nanometer range (Fig. 4).

### In vitro drug release study

**Fig. 5:**
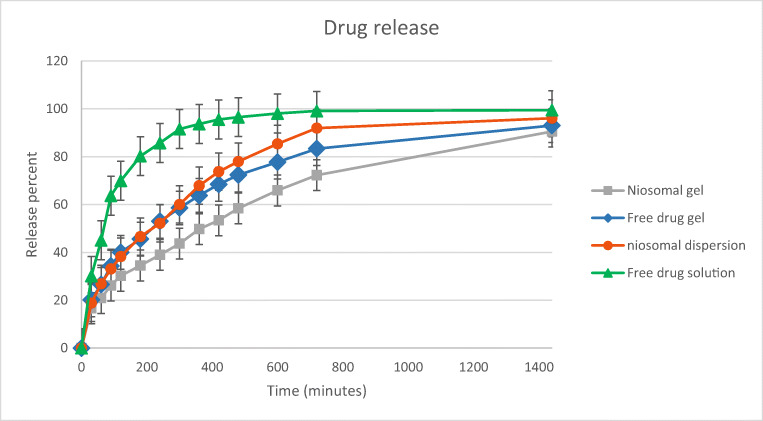
In vitro drug release profile of optimized niosomal dispersion, MB aqueous solution, and gel formulations

In vitro drug release profile of optimized niosomal dispersion, MB aqueous solution, and gel formulations are shown in Fig. 5. The Korsmeyer–Peppas release model showed the best fit (RSQ = 0.9987) in the optimized formulation compared with other models shown in Table 2. In this mathematical model, n is the release exponent which indicates the drug release mechanism. The n value of the optimized niosomal dispersion was found in the range of 0.43 and 0.85 indicating the drug release followed the non-Fickian (anomalous diffusion) mechanism, involving both diffusion and erosion [37]. On the other hand, the release data of the niosomal dispersion showed good fitting to the Higuchi release model (RSQ = 0.9973) and zero-order equation (RSQ = 0.9577). Due to the proper adaptation of the data in all three models. It can be concluded that the drug release from vesicles is more likely to occur through the diffusion process [38, 39]. In gel formulations containing niosomes or free drug, release data were best fitted in the Korsmeyer–Peppas model (RSQ equal to 0.9848 and 0.9906 respectively, 0.43 < *n* < 0.85) indicating the drug release followed non-Fickian diffusion. In the first hour, the cumulative percentage release of niosomal MB (26.897 ± 1.55%) was significantly lower than that of free drug (45.064 ± 0.93%) (*p* < 0.05). These results indicated that the vesicles can control the drug release rate and act as a prolonged drug delivery system compared with the free drug solution. These findings are in agreement with previous reports on the niosome potency for sustained drug release [40–42].

**Table 2:** Drug release kinetics data of optimized niosomal dispersion, MB aqueous solution, and gel formulations

	Zero order	First order	Korsmeyer–Peppas		Higuchi
	RSQ	RSQ	RSQ	*n*	RSQ
Niosomal dispersion	0.9577	0.8439	0.9987	0.5053	0.9973
Free drug solution	0.6892	0.5747	0.8973	–	0.8408
Niosomal gel	0.9452	0.8516	0.9848	0.4792	0.9811
Free drug gel	0.8928	0.7793	0.9906	0.474	0.9769

*RSQ*: linear regression coefficient; *n*: diffusional exponent in Korsmeyer–Peppas model; *β*: diffusional exponent in Weibull model

### FTIR analysis

**Fig. 6:**
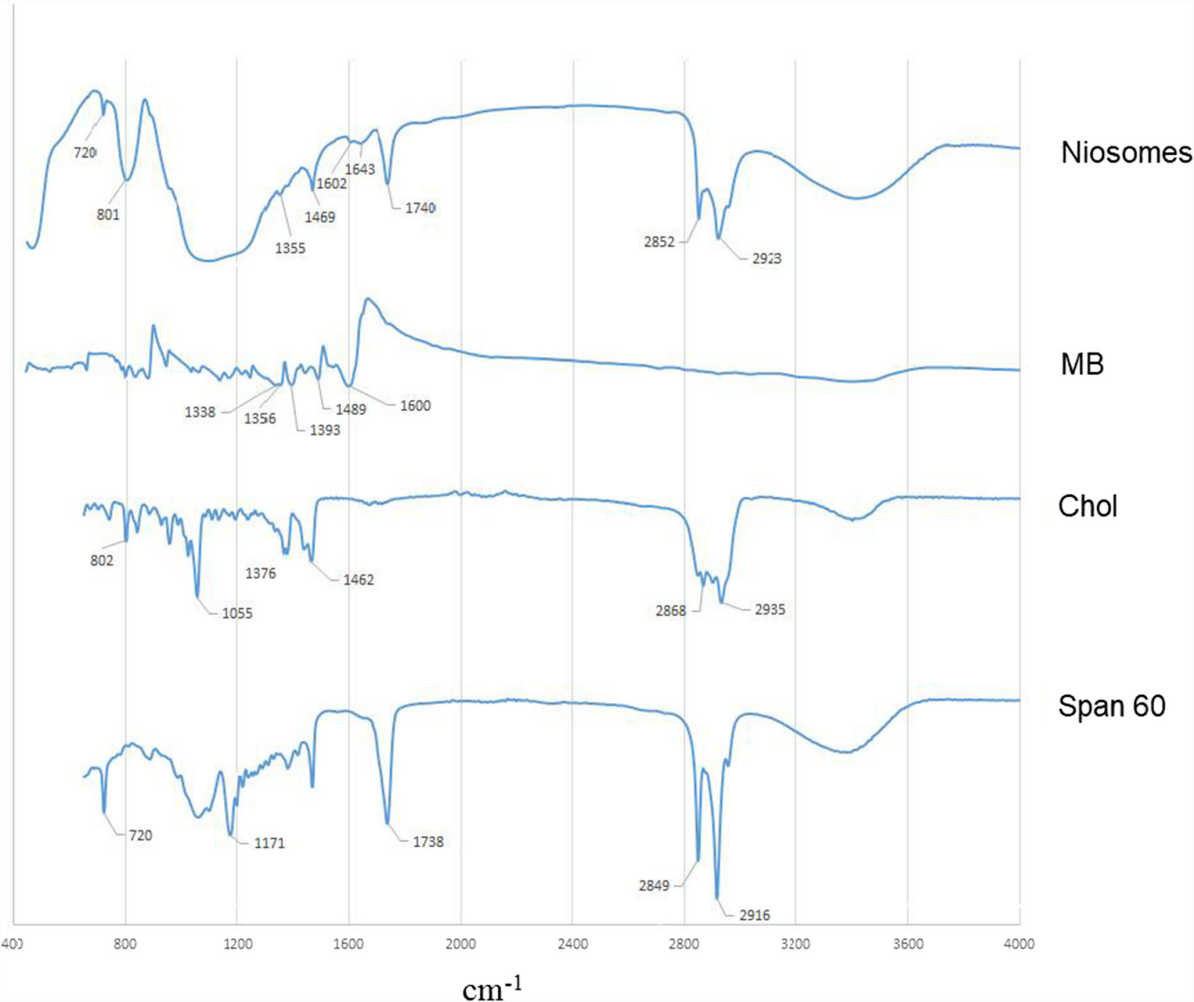
FTIR spectra of MB, Span 60, cholesterol, and freeze-dried niosome powder

The FTIR spectra of MB, Span 60, cholesterol, and freeze-dried niosome powder are shown in Fig. 6. The spectrum of MB showed characteristic peaks as C=C aromatic ring vibration (1600 cm^−1^), C–N stretching (1489 and 1393 cm^−1^) and methylene bending (1356 and 1338 cm^−1^). Spectrum of cholesterol displayed characteristic peaks of methylene rocking (802 cm^−1^), C–O stretching (1055 cm^−1^), C–H bond stretching (2800–3000 cm^−1^), C–H bond bending (1376 cm − 1), and –OH stretching (a broad peak in the range of 3100–3600 cm^−1^). Span 60 showed peaks as C=O stretching (1738 cm^−1^), –C–CO–O– (1171 cm^−1^), aliphatic CH stretching, asymmetric and symmetric (2916 cm^−1^ and 2849 cm^−1^ respectively), and aliphatic –CH2– rocking (722 cm^−1^). The characteristics peaks of MB, cholesterol, and Span 60 were presented in niosome FTIR spectra which confirmed the absence of any chemical interactions between them. A very broad peak which appeared in the range 3000–3700 cm^−1^ is attributed to a strong hydrogen bond between formulation components and according to previous studies, the hydrogen bond is most likely to form between cholesterol and Span 60 (Supplementary Fig. 3) [27].

### In vivo wound healing study

**Fig. 7:**
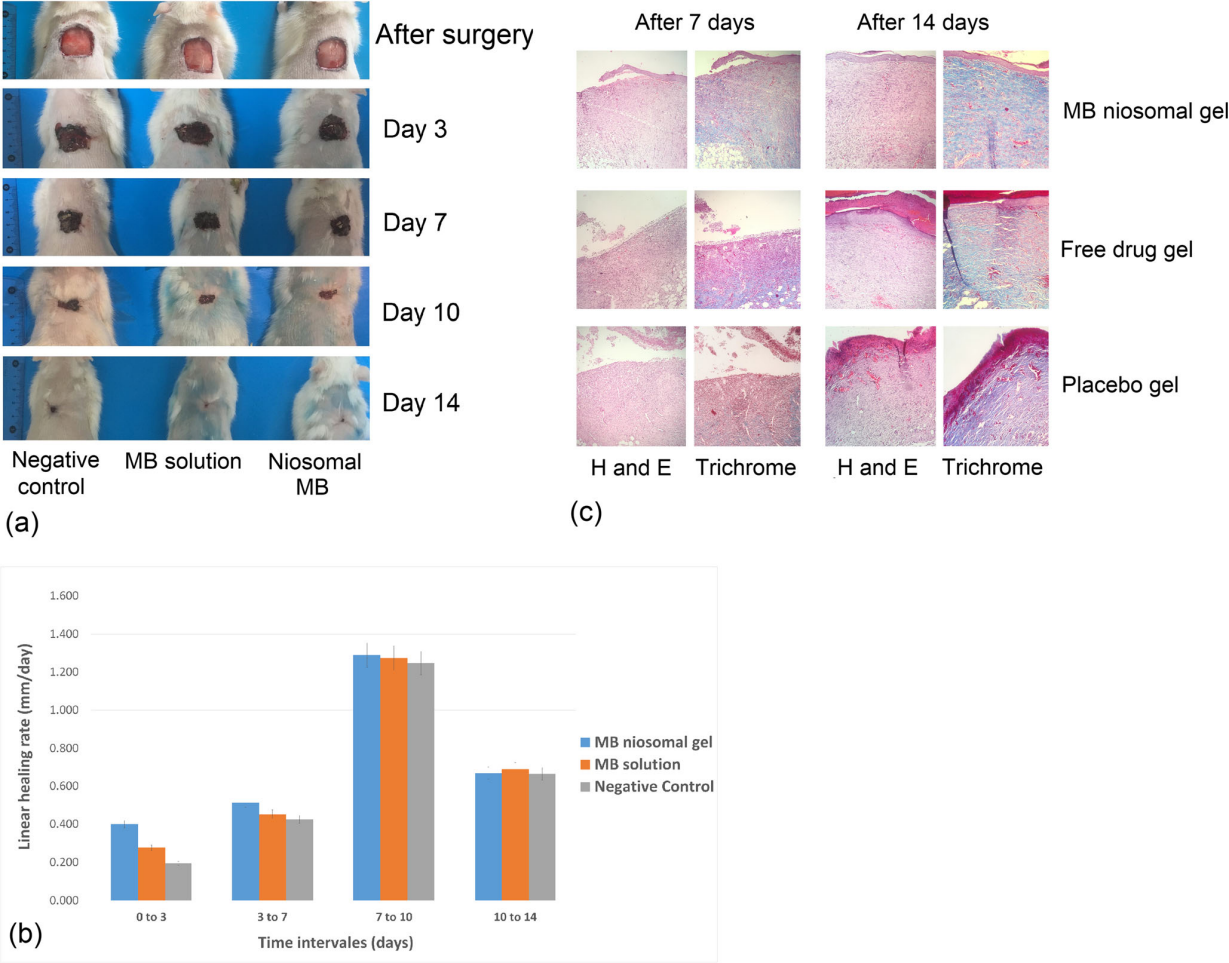
**a** Digital photographs of excision wounds healing process by MB niosomal gel, free drug gel, and placebo gel treatment on day 0, 3, 7, 10, and 14 after surgery. **b** Linear wound healing rate in different treatment groups at different time intervals. **c** Histological features of hematoxylin and eosin (H&E) and Masson’s trichrome (MT) of skin tissue samples of different treatment groups on days 7 and 14 after surgery

The optimized MB noisome formulation was converted into gel dosage form and applied transdermally for in vivo wound healing studies. As shown in Fig. 7a, by day 14 wounds treated with MB (niosomal or free drug gel) were approximately closed, while those treated with placebo gel remained slightly open. The linear wound healing rate was calculated at different time intervals. The results showed that the wound healing rate in the niosomal MB-treated group was higher than the other groups during the first week after surgery (*P* < 0.05) (Fig. 7b). The speed of recovery in the study groups was close to each other over time, and on day 14, there was no significant difference. The anti-inflammatory and antioxidant potential of MB accelerated the wound healing process [6, 8]. The histological evidence and tissue enzyme profile indicate that the wound healing progress is associated with MB antioxidant activity and the details will be discussed in the next sections.

### Histopathology

The repair of skin architecture has been studied by H&E and MT staining of the tissue specimens. After an injury, vascular endothelial growth factor (VEGF) and platelet-derived growth factor (PDGF), initiate angiogenesis and mediate blood vessel growth [43]. Angiogenesis is amplified during the inflammatory stage. In the proliferation phase, the number of newly formed vessels exceed the normal level. During the remodeling phase, many endothelial cells undergo apoptosis and the number of vessels decreases [44, 45]. Histopathological evaluation showed that 7 days after surgery, neovascularization in granulation tissue developed tended to be positively associated with inflammatory cells and fibroblasts. One week after surgery, new epithelium formed at the edge of the wounds in all of the three groups but developed in the treatment group (Fig. 7c). Collagen is the major structural protein in the extracellular matrix creating scaffolds for cellular attachment, growth, and differentiation [46]. In the remodeling stage, collagen reorganization occurs and the formation of cross-links between fibers improves wound tensile strength [47]. MT staining can highlight the collagen fiber remodeling (the protein is stained blue) [48]. After 7 days of treatment, the tissue samples stained with MT and niosomal gel-treated wounds showed higher collagen production compared with other groups (Fig. 7c). At day 14, there were thicker collagen fibers in organized parallel bundles, populated with less inflammatory infiltrate and more fibroblasts in niosomal gel treatment group. At the same time, thin granulation tissue, a considerable number of inflammatory cells, and disorganized collagen fibers were observed in wounds of both free MB-treated and control groups. As previously described, histological characteristic parameters were scored to compare healing speed between groups. Results showed that niosomal gel-treated wounds earned the highest scores in this comparison (Supplementary Fig. 4).

### Antioxidant status of wound tissue

**Fig. 8:**
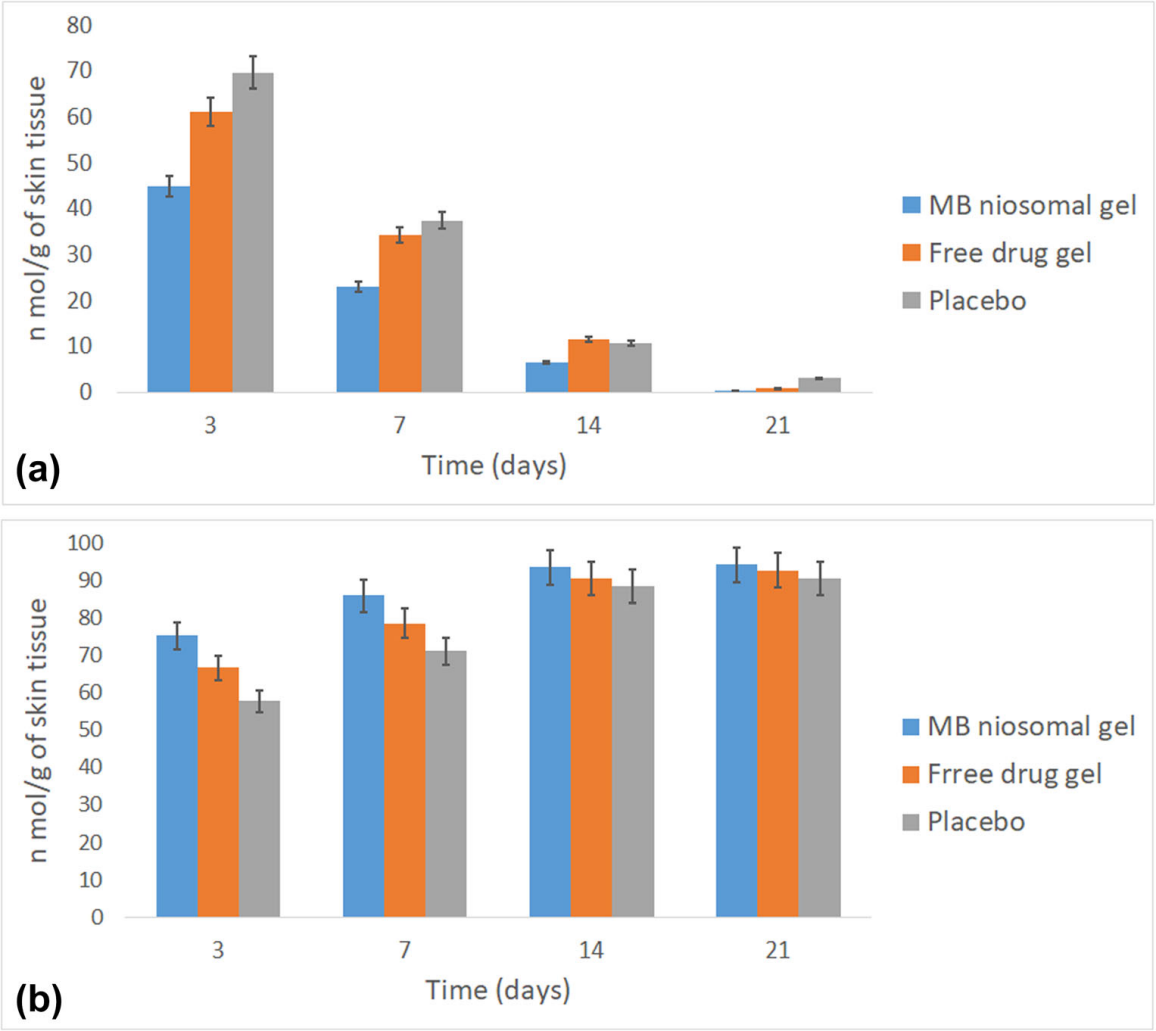
Malondialdehyde (**a**) and superoxide dismutase (**b**) levels in tissue homogenate of skin samples on days 3, 7, 14, and 21 after surgery

To determine the extent of lipid peroxidation, the MDA levels in tissue homogenate of skin samples were evaluated and the results are shown in Fig. 8a. On day 3 after surgery, the MDA level was significantly decreased in niosomal gel-treated rats compared with negative control and bulk-treated groups (*P* < 0.05). Similar results were found at day 7 post-surgery. The data showed no significant differences between the levels of MDA in the studied groups at days 14 and 21. The effect of bulk and niosomal MB on SOD activity in the skin samples is shown in Fig. 8b. The results of the SOD level measurement on the 3rd and 7th days after surgery showed that treatment with niosomal gel caused a significant inhibition in the SOD activity; consequently, the enzyme level was elevated compared with other groups (*p* < 0.05). MB is known as a redox dye, which increases the rate of cytochrome c reduction in mitochondria. The MB electron shunt effect reduces mitochondrial superoxide free radical production [6]. Thus, MB acts as an antioxidant and reduces cell damage during wound healing. Xiong et al. studied oxidative stress as a major cause of skin aging. Efficacy of MB on wound healing acceleration was evaluated using cultured 3D human skin model and the results showed an increase in the proliferation rate of fibroblasts. They concluded that MB promotes skin repair capabilities [7].

On day 14, levels of MDA restored to near normal in all groups. The wound healing process was found to be accelerated in the treatment group due to the decrease in the levels of antioxidants.

## Conclusion

In the present study, the Box–Behnken design was employed to achieve the maximum drug %EE and minimum vesicle size. The results showed that this statistical technique was efficient in optimizing the properties of nanoparticles. One of the main challenges of this study was the loading of a hydrophilic compound (MB) into the vesicles. The results showed that the optimized formulation had high encapsulation efficiency and good stability. Macroscopic, pathological, and biochemical studies of surgical wounds showed that the recovery rate in the niosomal MB-treated group was higher than the other groups. To evaluate the efficacy of niosomal gel in human wounds, the most important issue facing researchers is the sterilization of the product, which is possible by controlling the raw materials for the presence of microorganisms and pyrogen compounds and the production environment. The next issue is determining the appropriate dose of the drug. Sustained drug delivery reduces the need for multiple prescriptions and the proper penetration of niosomes into the skin tissue which reduces the amount of drug required per administration. In this study, MB dose was determined based on previous studies. It is suggested that in clinical studies, the dose of the drug should be selected as a variable to determine its optimal value for wound healing.

## Electronic supplementary material

ESM 1 (DOCX 384 kb)

## References

[1] Kim HS, Sun X, Lee J-H, Kim H-W, Fu X, Leong KW. Advanced drug delivery systems and artificial skin grafts for skin wound healing. Adv Drug Deliv Rev. 2018. 10.1016/j.addr.2018.12.014 30605737

[2] Rodero MP, Khosrotehrani K (2010). Skin wound healing modulation by macrophages. Int J Clin Exp Pathol.

[3] Liu WY, Tzeng T-F, Liu I-M (2017). Healing potential of zerumbone ointment on experimental full-thickness excision cutaneous wounds in rat. J Tissue Viability.

[4] Schäfer M, Werner S (2008). Oxidative stress in normal and impaired wound repair. Pharmacol Res.

[5] Rasik AM, Shukla A (2000). Antioxidant status in delayed healing type of wounds. Int J Exp Pathol.

[6] Atamna H, Nguyen A, Schultz C, Boyle K, Newberry J, Kato H, Ames BN (2008). Methylene blue delays cellular senescence and enhances key mitochondrial biochemical pathways. FASEB J.

[7] Xiong Z-M, O’Donovan M, Sun L, Choi JY, Ren M, Cao K (2017). Anti-aging potentials of methylene blue for human skin longevity. Sci Rep.

[8] Rosique MJ, Rosique RG, Faria FM, Oliveira CC, Farina JA, Évora PR (2017). Methylene blue reduces progression of burn and increases skin survival in an experimental rat model. Burns..

[9] Kotak K, Schulte A, Hay J, Sugden J (1997). Photostability of aniline blue (CI 42755) and methyl blue (CI 42780). Dyes Pigments.

[10] Schreier H, Bouwstra J (1994). Liposomes and niosomes as topical drug carriers: dermal and transdermal drug delivery. J Control Release.

[11] Azeem A, Anwer MK, Talegaonkar S (2009). Niosomes in sustained and targeted drug delivery: some recent advances. J Drug Target.

[12] Mozafari M, Pardakhty A, Azarmi S, Jazayeri J, Nokhodchi A, Omri A (2009). Role of nanocarrier systems in cancer nanotherapy. J Liposome Res.

[13] Moghassemi S, Hadjizadeh A (2014). Nano-niosomes as nanoscale drug delivery systems: an illustrated review. J Control Release.

[14] Uchegbu IF, Vyas SP (1998). Non-ionic surfactant based vesicles (niosomes) in drug delivery. Int J Pharm.

[15] Ge X, Wei M, He S, Yuan W-E (2019). Advances of non-ionic surfactant vesicles (niosomes) and their application in drug delivery. Pharmaceutics..

[16] Ghafourian T, Nokhodchi A, Kaialy W (2015). Surfactants as penetration enhancers for dermal and transdermal drug delivery. Percutaneous Penetration Enhancers Chemical Methods in Penetration Enhancement.

[17] Dragicevic N, Maibach HI (2016). Percutaneous penetration enhancers chemical methods in penetration enhancement.

[18] Arciero JC, Mi Q, Branca M, Hackam D, Swigon D (2013). Using a continuum model to predict closure time of gaps in intestinal epithelial cell layers. Wound Repair Regen.

[19] Vidal A, Zerón HM, Giacaman I, Romero MSC, López SP, Trillo LEM (2015). A Simple mathematical model for wound closure evaluation. J Am Coll Clin Wound Specialists.

[20] Muhammad AA, Arulselvan P, Cheah PS, Abas F, Fakurazi S (2016). Evaluation of wound healing properties of bioactive aqueous fraction from *Moringa oleifera* Lam on experimentally induced diabetic animal model. Drug Des Devel Ther.

[21] Uchiyama M, Mihara M (1978). Determination of malonaldehyde precursor in tissues by thiobarbituric acid test. Anal Biochem.

[22] Marklund SL. Pyrogallol autooxidation. Handbook of methods for oxygen radical research. 1985;243:247.

[23] Walker I, Gorman SA, Cox RD, Vernon DI, Griffiths J, Brown SB (2004). A comparative analysis of phenothiazinium salts for the photosensitisation of murine fibrosarcoma (RIF-1) cells in vitro. Photochem Photobiol Sci.

[24] Junyaprasert VB, Singhsa P, Suksiriworapong J, Chantasart D (2012). Physicochemical properties and skin permeation of Span 60/Tween 60 niosomes of ellagic acid. Int J Pharm.

[25] Bayindir ZS, Yuksel N (2010). Characterization of niosomes prepared with various nonionic surfactants for paclitaxel oral delivery. J Pharm Sci.

[26] Bnyan R, Khan I, Ehtezazi T, Saleem I, Gordon S, O'Neill F, Roberts M (2018). Surfactant effects on lipid-based vesicles properties. J Pharm Sci.

[27] Nasseri B (2005). Effect of cholesterol and temperature on the elastic properties of niosomal membranes. Int J Pharm.

[28] Sezgin-Bayindir Z, Yuksel N (2012). Investigation of formulation variables and excipient interaction on the production of niosomes. AAPS PharmSciTech.

[29] Essa EA. Effect of formulation and processing variables on the particle size of sorbitan monopalmitate niosomes. Asian J Pharm. 2014;4(4).

[30] Arafa MG, Ayoub BM (2017). DOE optimization of nano-based carrier of pregabalin as hydrogel: new therapeutic & chemometric approaches for controlled drug delivery systems. Sci Rep.

[31] Mukhopadhyay L, Bhattacharyya P, Das A, Moulik S (1993). Surfactant stabilised colloidal cholesterol. Colloid Polym Sci.

[32] Magarkar A, Dhawan V, Kallinteri P, Viitala T, Elmowafy M, Róg T (2014). Cholesterol level affects surface charge of lipid membranes in saline solution. Sci Rep.

[33] Rager T, Geoffroy A, Hilfiker R, Storey JM (2012). The crystalline state of methylene blue: a zoo of hydrates. Phys Chem Chem Phys.

[34] Peltonen L. The interfacial behaviour of sorbitan surfactant monolayers and the bulk properties of these surfactants as a function of temperature. 2001.

[35] Uskoković V (2008). Insights into morphological nature of precipitation of cholesterol. Steroids..

[36] Milošević MD, Logar MM, Poharc-Logar AV, Jakšić NL. Orientation and optical polarized spectra (380–900 nm) of methylene blue crystals on a glass surface. Int J Spectrosc. 2013;2013.

[37] Siepmann J, Siepmann F (2008). Mathematical modeling of drug delivery. Int J Pharm.

[38] Srinivas S, Kumar YA, Hemanth A, Anitha M (2010). Preparation and evaluation of niosomes containing aceclofenac. Dig J Nanomater Biostruct.

[39] Alemi A, Reza JZ, Haghiralsadat F, Jaliani HZ, Karamallah MH, Hosseini SA (2018). Paclitaxel and curcumin coadministration in novel cationic PEGylated niosomal formulations exhibit enhanced synergistic antitumor efficacy. J Nanobiotechnol.

[40] Abdelbary G, El-gendy N (2008). Niosome-encapsulated gentamicin for ophthalmic controlled delivery. AAPS PharmSciTech.

[41] Tavano L, Muzzalupo R, Picci N, de Cindio B (2014). Co-encapsulation of antioxidants into niosomal carriers: gastrointestinal release studies for nutraceutical applications. Colloids Surf B: Biointerfaces.

[42] Moghassemi S, Hadjizadeh A, Hakamivala A, Omidfar K (2017). Growth factor-loaded nano-niosomal gel formulation and characterization. AAPS PharmSciTech.

[43] Li WW, Tsakayannis D, Li VW (2003). Angiogenesis: a control point for normal and delayed wound healing. Contemp Surg.

[44] DiPietro LA (2016). Angiogenesis and wound repair: when enough is enough. J Leukoc Biol.

[45] Beyer S, Koch M, Lee Y, Jung F, Blocki A (2018). An in vitro model of angiogenesis during wound healing provides insights into the complex role of cells and factors in the inflammatory and proliferation phase. Int J Mol Sci.

[46] Ruszczak Z (2003). Effect of collagen matrices on dermal wound healing. Adv Drug Deliv Rev.

[47] Xue M, Jackson CJ (2015). Extracellular matrix reorganization during wound healing and its impact on abnormal scarring. Adv Wound Care.

[48] Wang W, Lin S, Xiao Y, Huang Y, Tan Y, Cai L (2008). Acceleration of diabetic wound healing with chitosan-crosslinked collagen sponge containing recombinant human acidic fibroblast growth factor in healing-impaired STZ diabetic rats. Life Sci.

