# Supplementary material for: Methylene blue-loaded niosome: preparation, physicochemical characterization, and in vivo wound healing assessment
Source: Drug Deliv Transl Res. 2020 Feb 25;10(5):1428–41. doi: 10.1007/s13346-020-00715-6 (PMC7447683; doi:10.1007/s13346-020-00715-6)
Supplement: Supplementary file 1 — (DOCX 384 kb) [file 13346_2020_715_MOESM1_ESM.docx]

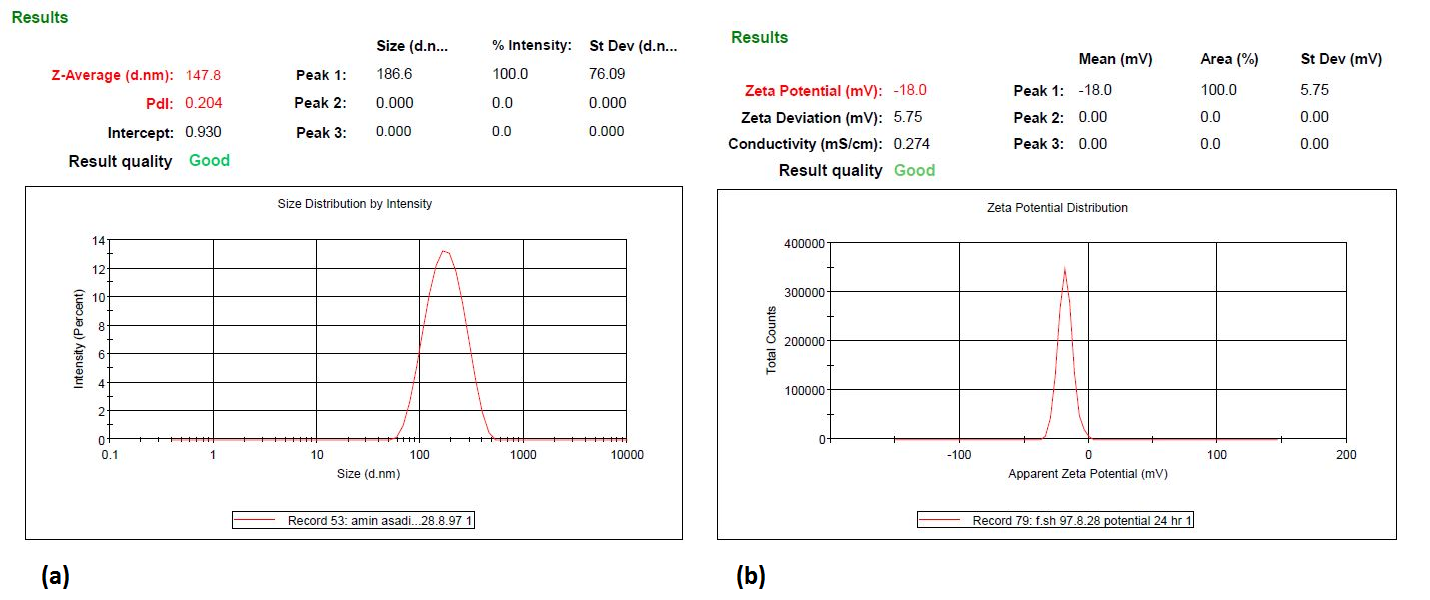


Supplementary Fig 1. Observed response values for the predicted optimal formulation.


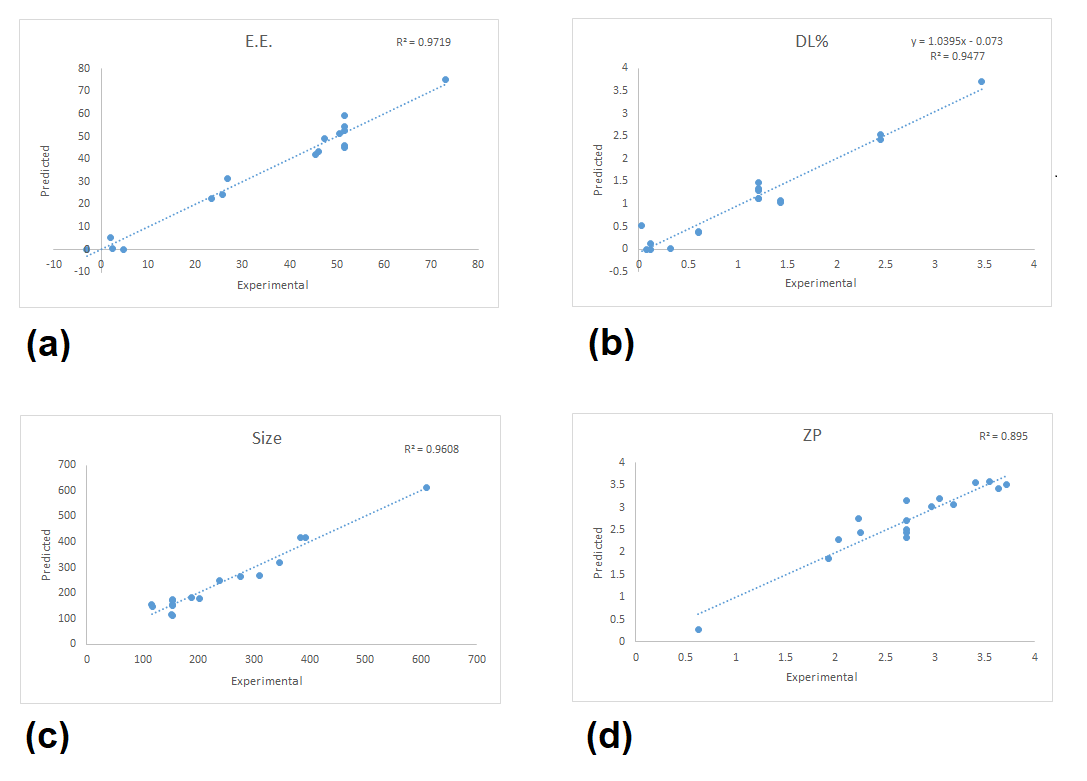


Supplementary Fig 2. Relationship between predicted and experimental values of all dependent variables. (a) encapsulation efficiency percent (E.E.%), (b) drug loading percent (DL%), (c) particle size, and (d) Zeta potential (ZP).


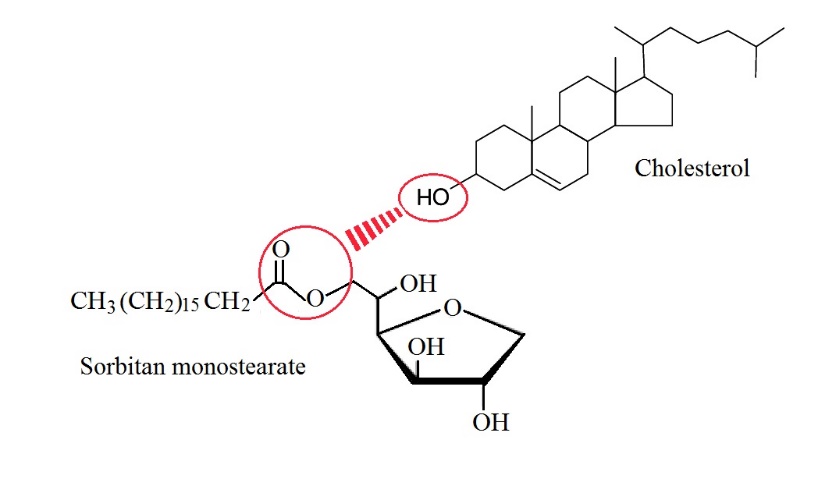


Supplementary Fig 3. The possible hydrogen bonding interaction between the cholesterol and Tween 60

Supplementary Fig 4. Histomorphologic scores of incisional wounds at 7 and 14 days after surgery.
